# Global, regional and national trends and impacts of natural floods, 1990–2022

**DOI:** 10.2471/BLT.23.290243

**Published:** 2024-04-17

**Authors:** Qiao Liu, Min Du, Yaping Wang, Jie Deng, Wenxin Yan, Chenyuan Qin, Min Liu, Jue Liu

**Affiliations:** a Department of Epidemiology and Biostatistics, School of Public Health, Peking University, No. 38, Xueyuan Road, Haidian District, Beijing 100191, China.

## Abstract

**Objective:**

To assess global, regional and national trends in the impact of floods from 1990 to 2022 and determine factors influencing flood-related deaths.

**Methods:**

We used data on flood disasters from the International Disaster Database for 1990–2022 from 168 countries. We calculated the annual percentage change to estimate trends in the rates of people affected and killed by floods by study period, World Health Organization (WHO) region, country income level and flood type. We used multivariable logistic regression analysis to assess the factors associated with death from floods.

**Findings:**

From 1990 to 2022, 4713 floods were recorded in 168 countries, which affected > 3.2 billion people, caused 218 353 deaths and were responsible for more than 1.3 trillion United States dollars of economic losses. The WHO Western Pacific Region had the most people affected by floods (> 2.0 billion), accounting for 63.19% (2 024 599 380/3 203 944 965) of all affected populations. The South-East Asia Region had the most deaths (71 713, 32.84%). The African and Eastern Mediterranean Regions had the highest number of people affected and killed by floods per 100 000 population in 2022. The odds of floods causing more than 50 deaths were significantly higher in low-income countries (adjusted odds ratio: 14.34; 95% confidence interval: 7.46 to 30.04) compared with high-income countries. Numbers of people affected and mortality due to floods declined over time.

**Conclusion:**

Despite the decreases in populations affected and deaths, floods still have a serious impact on people and economies globally, particularly in lower-income countries. Action is needed to improve disaster risk management and flood mitigation.

## Résumé

**Objectif:**

Évaluer les tendances mondiales, régionales et nationales relatives à l'impact des inondations entre 1990 et 2022, mais aussi déterminer les facteurs influençant les décès causés par ces inondations.

**Méthodes:**

Nous avons exploité les données concernant 168 pays et reprises dans la base de données internationale sur les catastrophes pour la période allant de 1990 à 2022. Nous avons ensuite calculé la variation annuelle en pourcentage afin de dégager des tendances quant au nombre de personnes touchées, voire tuées par des inondations en fonction de la période d'étude, de la région telle que définie par l'Organisation mondiale de la Santé (OMS), du niveau de revenu du pays et du type d'inondation. Enfin, nous avons employé une analyse multivariée de régression logistique pour déterminer les facteurs associés aux décès en cas d'inondations.

**Résultats:**

Entre 1990 et 2022, 4713 inondations ont été recensées dans 168 pays. Elles ont touché plus de 3,2 milliards de personnes, causé 218 353 morts et entraîné des pertes économiques supérieures à 1,3 billion de dollars américains. La région du Pacifique occidental de l'OMS est celle où le plus grand nombre d'habitants ont été affectés par ces phénomènes (> 2 milliards), représentant 63,19 % (2 024 599 380 / 3 203 944 965) de l'ensemble des populations impactées. C'est la région d'Asie du Sud-Est qui comptait le plus grand nombre de décès (71 713, soit 32,84 %). La région Africaine et la région de la Méditerranée orientale dénombraient toutes deux la plus grande proportion de personnes touchées et tuées lors d'inondations par 100 000 habitants en 2022. La probabilité que les inondations soient à l'origine de plus de 50 morts était nettement plus importante dans les pays à revenu faible (odds ratio ajusté : 14,34 ; intervalle de confiance à 95 % : 7,46 à 30,04) que dans les pays à revenu élevé. L'étendue des répercussions et le taux de mortalité des inondations diminuaient au fil du temps.

**Conclusion:**

Malgré une baisse du nombre de personnes touchées et tuées, les inondations continuent à avoir un impact considérable sur les populations et les économies à travers le monde, surtout dans les pays aux revenus plus faibles. Des actions sont requises pour améliorer la gestion des risques de catastrophes et la lutte contre les inondations.

## Resumen

**Objetivo:**

Evaluar las tendencias mundiales, regionales y nacionales del impacto de las inundaciones de 1990 a 2022 y determinar los factores que influyen en las muertes relacionadas con las inundaciones.

**Métodos:**

Se utilizaron datos sobre catástrofes por inundaciones de la Base de Datos Internacional sobre catástrofes para 1990-2022 de 168 países. Se calculó la variación porcentual anual para estimar las tendencias de las tasas de personas afectadas y fallecidas por inundaciones según el periodo de estudio, la región de la Organización Mundial de la Salud (OMS), el nivel de ingresos del país y el tipo de inundación. Se utilizó un análisis de regresión logística multivariable para evaluar los factores asociados a la muerte por inundaciones.

**Resultados:**

Entre 1990 y 2022, se registraron 4713 inundaciones en 168 países, que afectaron a >3200 millones de personas, causaron 218 353 muertes y fueron responsables de más de 1,3 billones de dólares estadounidenses de pérdidas económicas. La Región del Pacífico Occidental de la OMS fue la más afectada por las inundaciones (>2000 millones), con un 63,19% (2 024 599 380/3 203 944 965) de todas las poblaciones afectadas. La Región de Asia Sudoriental fue la que registró más muertes (71 713, 32,84%). Las regiones de África y el Mediterráneo Oriental registraron el mayor número de personas afectadas y fallecidas por inundaciones por cada 100 000 habitantes en 2022. Las probabilidades de que las inundaciones causaran más de 50 muertes fueron significativamente mayores en los países de ingresos bajos (razón de probabilidades ajustada: 14,34; intervalo de confianza del 95%: 7,46 a 30,04) en comparación con los países de ingresos altos. El número de personas afectadas y la mortalidad por inundaciones disminuyeron con el tiempo.

**Conclusión:**

A pesar de la disminución de las poblaciones afectadas y de las muertes, las inundaciones siguen teniendo un grave impacto en las personas y las economías de todo el mundo, en especial en los países con ingresos más bajos. Es preciso actuar para mejorar la gestión del riesgo de catástrofes y la mitigación de las inundaciones.

## ملخص

**الغرض:**

تقييم الاتجاهات العالمية والإقليمية والوطنية في تأثير الفيضانات للفترة من 1990 إلى 2022، وتحديد العوامل المؤثرة على الوفيات المرتبطة بالفيضانات.

**الطريقة:**

قمنا باستخدام البيانات الخاصة بكوارث الفيضانات من قاعدة بيانات الكوارث الدولية للفترة من 1990 إلى 2022، من 168 دولة. وقمنا بحساب التغيُّر في النسبة المئوية لتقدير الاتجاهات في معدلات الأشخاص المتضررين والمتوفين بسبب الفيضانات، وذلك حسب فترة الدراسة، ومنطقة منظمة الصحة العالمية (WHO)، ومستوى الدخل في الدولة، ونوع الفيضان. كما استخدمنا تحليل التحوّف اللوجستي متعدد المتغيرات لتقييم العوامل المرتبطة بالوفيات المترتبة على الفيضانات.

**النتائج:**

في الفترة من 1990 إلى 2022، تم تسجيل 4713 فيضانًا في 168 دولة، مما أثر على أكثر من 3.2 مليار شخص، وتسبب في وفاة 218353 شخصًا، وكانت مسؤولة عن خسائر اقتصادية تزيد قيمتها عن 1.3 تريليون دولار أمريكي. سجّل إقليم غرب المحيط الهادئ التابع لمنظمة الصحة العالمية أكبر عدد من الأشخاص المتضررين بسبب الفيضانات (أكثر من 2.0 مليار شخص)، وهو ما يمثل %63.19 (2024599380/3203944965) من جميع السكان المتضررين. وشهدت منطقة جنوب شرق آسيا أكبر عدد من الوفيات (71713، %32.84). وسجّلت منطقتا أفريقيا وشرق البحر الأبيض المتوسط أكبر عدد من الأشخاص المتضررين والوفيات بسبب الفيضانات لكل 100000 نسمة في عام 2022. وكانت احتمالات تسبب الفيضانات في أكثر من 50 حالة وفاة أعلى بشكل ملموس في الدول ذات الدخل المنخفض (نسبة الاحتمالات المعدلة: 14.34؛ وبفاصل ثقة مقداره %95: 7.46؛ %95) مقارنة بالدول ذات الدخل المرتفع. انخفض معدل التأثير وعدد الوفيات الناتجة عن الفيضانات مع مرور الوقت.

**الاستنتاج:**

على الرغم من حالات الانخفاض عدد السكان المتضررين والوفيات، لا تزال الفيضانات ذات تأثير خطير على الأشخاص والنظم الاقتصادية على مستوى العالم، وبخاصة في الدول ذات الدخل المنخفض. هناك حاجة إلى اتخاذ إجراءات لتحسين إدارة مخاطر الكوارث، والتخفيف من تأثير الفيضانات.

## 摘要

**目的:**

旨在评估 1990 年至 2022 年期间洪水在全球、区域和国家层面的影响趋势，并确定影响洪水致死率的因素。

**方法:**

我们利用从国际灾难数据库获取的 1990-2022 年期间 168 个国家的洪水灾害相关数据。我们计算了年度百分比变化，从而基于研究时段、世界卫生组织 (WHO) 区域、国家收入水平和洪水类型估计了因洪水而受影响甚至致死的人数比例变化趋势。我们使用了多变量逻辑回归分析方法来评估洪水致死率相关因素。

**结果:**

记录显示，1990 年至 2022 年期间，168 个国家共发生了 4,713 次洪水，超过 32 亿人受到影响，导致 218,353 人死亡，造成经济损失超过了 1.3 万亿美元。WHO 西太平洋区域受洪水影响的人数最多（超过 20 亿），占所有受影响人口的 63.19% (2,024,599,380/3,203,944,965)。东南亚区域因洪水致死的人数最多（71,713 人，占 32.84%）。2022 年非洲和东地中海区域每 100,000 人口中因洪水而受影响甚至致死的人数最多。与高收入国家相比，低收入国家因洪水造成 50 人以上死亡的几率要高得多（调整后优势比：14.34；95% 置信区间：7.46 至 30.04）。随着时间的推移，洪水的影响率和死亡率均有所下降。

**结论:**

尽管受影响人口和致死人数均有所减少，但洪水仍然严重影响着全球人类和全球经济，特别是在低收入国家。需要采取行动以加强灾害风险管理和减轻洪灾影响。

## Резюме

**Цель:**

Оценить глобальные, региональные и национальные тенденции в отношении последствий наводнений с 1990 по 2022 год и определить факторы, влияющие на смертность от наводнений.

**Методы:**

В статье использованы данные о наводнениях из Международной базы данных о чрезвычайных ситуациях за 1990–2022 гг. по 168 странам. Чтобы оценить тенденции изменения числа пострадавших и погибших от наводнений, авторы рассчитали годовое процентное изменение в зависимости от периода исследования, региона Всемирной организации здравоохранения (ВОЗ), уровня дохода в стране и типа наводнения. Для оценки факторов, связанных со смертью от наводнений, использовался многомерный логистический регрессионный анализ.

**Результаты:**

За период с 1990 по 2022 год в 168 странах было зарегистрировано 4713 случаев наводнений, в результате которых пострадали более 3,2 миллиарда человек, погибли 218 353 человека, а экономический ущерб составил более 1,3 триллиона долларов США. Больше всего пострадавших от наводнений (> 2,0 млрд человек) пришлось на Западно-Тихоокеанский регион ВОЗ – 63,19% (2 024 599 380/3 203 944 965) от общей численности пострадавшего населения. Больше всего случаев летального исхода было зарегистрировано в регионе Юго-Восточной Азии (71 713, 32,84%). В 2022 году наибольшее число пострадавших и погибших от наводнений на 100 000 человек населения приходится на Африканский и Восточно-Средиземноморский регионы. Вероятность того, что в результате наводнений погибнет более 50 человек, была значительно выше в странах с низким уровнем дохода (скорректированное отношение шансов: 14,34; 95%-й ДИ: 7,46–30,04) по сравнению со странами с высоким уровнем дохода. С течением времени количество пострадавших и смертность от наводнений снижались.

**Вывод:**

Несмотря на сокращение числа пострадавших и погибших, наводнения по-прежнему оказывают серьезное воздействие на людей и экономику во всем мире, особенно в странах с низким уровнем дохода. Необходимо принять меры для повышения эффективности управления рисками стихийных бедствий и смягчения последствий наводнений.

## Introduction

Natural disasters are catastrophic events of atmospheric, geological and hydrological origins. These disasters include earthquakes, floods, volcanic eruptions, landslides, tsunamis and droughts.^1^ From 1998 to 2017, 1.3 million people were reported to have died from climate-related and geophysical disasters, and a further 4.4 billion people were injured, made homeless, displaced or in need of emergency assistance.^2^ In the same period, disaster-hit countries also reported direct economic losses valued at 2.9 trillion United States dollars (US$).^2^ Disasters are a major contributor to entrenched poverty in low- and middle-income countries; thus, reducing losses from disasters is key to eradicating poverty. The United Nations (UN) Office for Disaster Risk Reduction called for a substantial reduction in direct disaster losses as well as associated infectious diseases, and social and economic losses, and noted that the responsibility should be shared by the stakeholders.^29^

Natural floods (hereafter called floods) are the most common natural hazard event and are the leading cause of death from disasters worldwide as well as serious health, social and economic consequences.^3^^–^^5^ A 2013 study showed that from 1980 to 2009, floods were responsible for more than 500 000 deaths and more than 350 000 injuries, and affected nearly 3 billion people across the world.^3^ The World Health Organization (WHO) South-East Asia and Western Pacific Regions were the most flood-affected regions and were estimated to account for nearly 50% of flood-related deaths in the last 25 years of the 20th century.^6^^,^^7^ Additionally, an estimated 1.81 billion people were directly exposed to 1-in-100-year floods, 1.24 billion of whom lived in the WHO South-East Asia Region.^8^ In the WHO European Region, about 400 floods caused the deaths of more than 2000 people, affected 8.7 million others, and resulted in at least 72 billion euros in losses during 2000–2014.^9^

A recent systematic review identified various factors that can influence flood-related deaths, which could be classified into five categories, namely hazard-related, individual, environmental, socioeconomic and managerial factors.^10^ However, which of these factors has a significant causal relationship with flood-related deaths is still unclear. Furthermore, the trends in the global impact of floods in the past three decades is not well understood. In this study, we aimed to summarize the trends in the global, regional and national impact of floods from 1990 to 2022, and to assess the factors associated with deaths from floods. The findings could provide a comprehensive understanding of the trends in the impact of floods and the factors influencing flood-related deaths. Such information is important to help strengthen disaster risk management and enhance preparedness for and effective responses to floods.

## Methods

### Study design and data sources

We conducted a worldwide observational study which covered all countries and territories that reported flood disasters from 1990 to 2022. Disaster-related data were extracted from the International Disaster Database (EM-DAT).^11^ This database was launched in 1988 by the Centre for Research on the Epidemiology of Disasters with the support of WHO and the Belgian government. The main objective of the database is to facilitate humanitarian action at national and international levels, rationalize decision-making for disaster preparedness, and provide an objective base for vulnerability assessment and priority-setting.^11^ EM-DAT contains essential core data on the occurrence and effects of different kinds of disasters in the world from 1900 to the present day. These data are compiled from various sources, including UN agencies, nongovernmental organizations, insurance companies, research institutes and press agencies.^11^ The income level and number of populations of various countries in each year from 1990 to 2022 were extracted from the UN open database, UNdata.^28^ No ethics approval was required for this analysis of publicly available data. 

### Variables

Flood was defined as the overﬂow of water from a stream channel onto normally dry land in the ﬂoodplain (riverine ﬂooding), higher-than-normal levels along the coast and by lakes or reservoirs (coastal ﬂooding), as well as ponding of water at or near the point where rain fell (ﬂash ﬂooding).^11^ In the EM-DAT, flood disasters are one of the hydrological disasters (including flood, landslide and wave action), which are defined as hazards caused by the occurrence, movement and distribution of surface and subsurface freshwater and saltwater. We used the following variables from the database: country (countries in which the flood occurred); date (when the flood occurred and ended); flood type (riverine ﬂooding, coastal ﬂooding or ﬂash ﬂooding); deaths (number of people who died because of the flood); affected individuals (number of people who were injured or made homeless by the flood or who required immediate assistance during a period of emergency, i.e. basic survival needs such as food, water, shelter, sanitation and immediate medical assistance); and adjusted total damages in US$ (all damages and economic losses directly or indirectly related to the flood, adjusted for inflation using the consumer price index).

### Statistical analysis

In the descriptive analysis, the number of affected people, the number of deaths and the total economic damages in each year were summed from 1990 to 2022 by country. The number of floods in each WHO region every month was also summed from 1990 to 2022. Given differences in population size and fluctuations of population size in different years and regions, we calculated the rates of people affected by flood per 100 000 population (affected rate) and the rates of flood-related deaths per 100 000 population (mortality) using the total number of people affected by the floods, the number of deaths caused by the floods, and the total population of each region in each year. We calculated the estimated annual percentage change to show trends. This indicator is widely used to show the trend and annual change over a specified time period.^12^^,^^13^ We fitted a regression line to the natural logarithm of rates using the formula *y* = *α* + *βx* + *ε*, where *y* is the rate, *x* is the calendar year, *α* is the expected value of the dependent variable when all explanatory variables are 0, *β* is regression coefficient and *ε* is the error term (the part of the *y* variable that the model cannot explain). We calculated the estimated annual percentage change, with its 95% confidence intervals (CIs) to assess the time trend in the rates, as 100 × (*e^β^* – 1), where *e* is the Euler number. If the estimated annual percentage change and its 95% CI were both > 0, we considered the trend in rates to be increasing in the given time interval. Conversely, if the change and 95% CI were both < 0, we considered the trend in rates to be decreasing.

Additionally, we divided all the flood events into four groups based on the number of deaths they caused: (i) zero deaths or no data; (ii) 1–9 deaths; (iii) 10–49 deaths; and (iv) ≥ 50 deaths. We used the *χ^2^* test to assess differences between the four groups by study period, WHO region, country income level and subtype of flood. We used multivariable logistic regression analysis to estimate the associations of factors (study period, WHO region, income level and flood type) with the risk of death at the group level compared with floods with 0 deaths or no data, expressed as adjusted odds ratios (aOR). We used R, version 4.1.1 (R Foundation, Vienna, Austria) for all analyses.

## Results

### Global and national impact

**Fig. 1:**
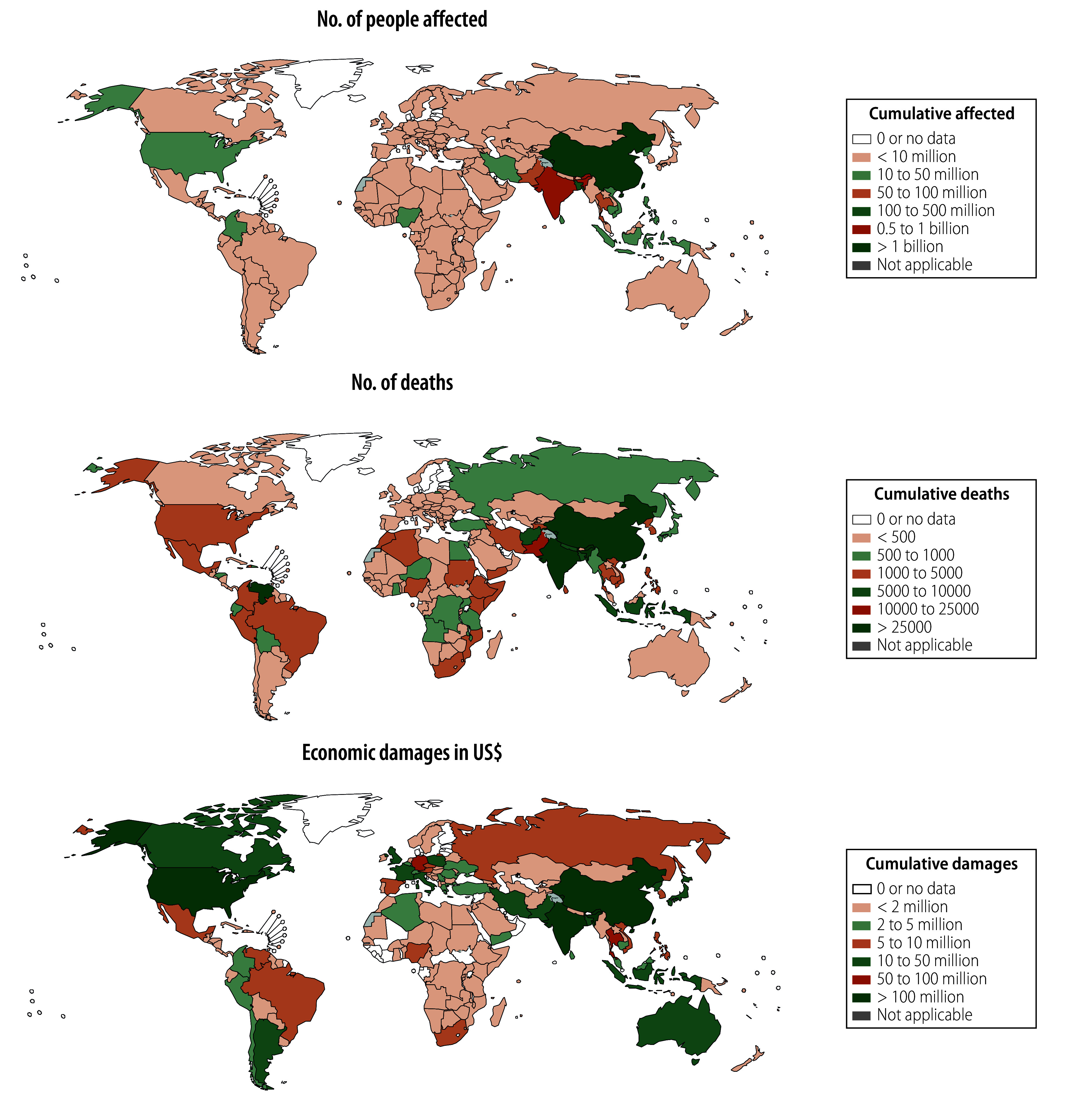
Cumulative impact of natural floods by country, 1990–2022

From 1990 to 2022, 4713 floods were recorded in 168 countries and territories, which affected more than 3.2 billion people, resulted in 218 353 deaths and caused more than US$ 1.3 trillion in economic damages. Of all the countries where floods occurred, China was the most affected, with the largest cumulative population affected (1.9 billion people), the most economic damage (US$ 442 billion) and the second largest number of deaths due to the floods (30 890 deaths). India had the second largest population affected (629 million people), the third most economic damages (US$ 115 billion) and the largest number of deaths (46 506 deaths). In Bangladesh, 159 million people were affected by floods, the third largest in the world. In the United States of America, floods caused about US$ 135 billion in economic damages, second only to China; and Bolivarian Republic of Venezuela recorded the third highest number of deaths (30 342 deaths) from floods (Fig. 1).

US$: United States dollars.

### Floods by WHO region

**Fig. 2:**
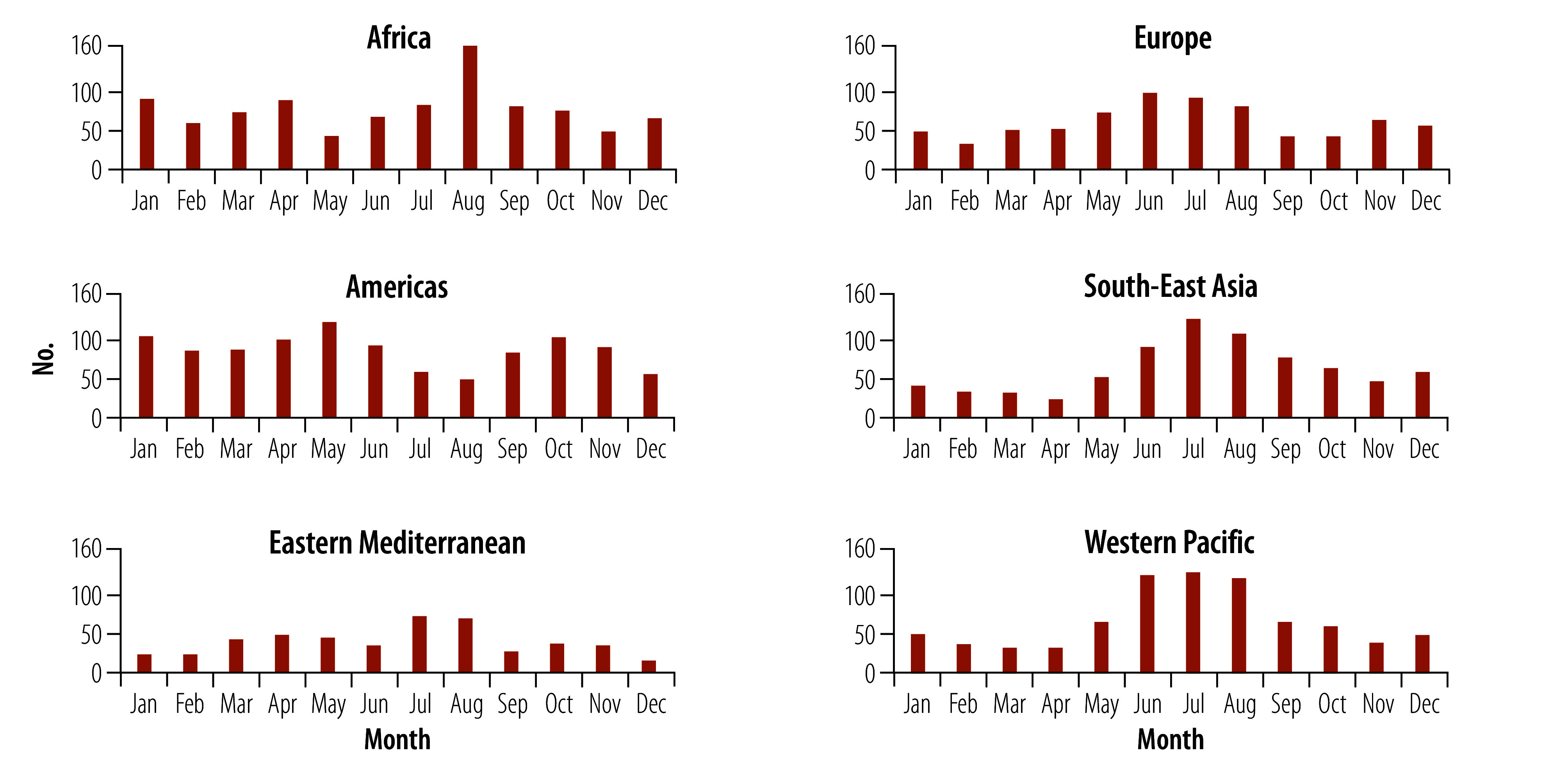
Number of natural floods per month, by WHO region, 1990–2022

Of the 4713 floods recorded, 930 (19.73%) occurred in the African Region; 1029 (21.83%) in the Regions of the Americas; 777 (16.49%) in the South-East Asia Region; 708 (15.02%) in the European Region; 485 (10.29%) in the Eastern Mediterranean Region; and 784 (16.63%) in the Western Pacific Region. In the African Region, the number of floods was highest in August (158 floods; 16.99%). In the Region of the Americas, the greatest number of floods occurred in May (122 floods; 11.86%), followed by 104 floods (10.11%) in January and 103 floods (10.01%) in October. In the European Region, floods mostly occurred from May to August, accounting for 47.60% (337/708) of the total floods. In the Eastern Mediterranean Region, most floods occurred in July and August, accounting for 29.28% (142/485) of the total floods. In both the South-East Asia and Western Pacific Regions, floods mostly occurred during June to September, accounting for 52.90% (411/777) and 54.97% (431/784) of the total floods, respectively (Fig. 2).

WHO: World Health Organization.

From 1990 to 2022, most people affected by floods were residing in the Western Pacific Region (> 2 billion people), representing 63.19% of all the affected populations (2 024 599 380/3 203 944 965), followed by 890 683 355 (27.80%) affected people living in the South-East Asia Region. The European Region had the lowest number of people affected by floods from 1990 to 2022 (17 million). In Western Pacific Region, there were 24 years when more than 10 million people were affected by floods yearly and, among those years, 10 years when more than 100 million people were affected. In the South-East Asia Region, there were 25 years when more than 10 million people were affected by floods yearly, and in 1993, 146 million people were affected. More than 10 million people were affected by floods in 4 years in the Eastern Mediterranean Region: 34 million in 2022, 21 million in 2010, 13 million in 1992 and 12 million in 2019. The highest number of people affected by floods in the Region of the Americas was 16 million in 2008 (Table 1). Globally, the largest number of people affected was in 1998 (243 million people), followed by 212 million people in 1991 and 157 million people in 1996.

**Table 1:** Number of affected people and deaths caused by natural floods, by year and WHO region, 1990–2022

Year	No. of people affected							No. of deaths					
	Africa	Americas	South-East Asia	Europe	Eastern Mediterranean	Western Pacific		Africa	Americas	South-East Asia	Europe	Eastern Mediterranean	Western Pacific
1990	540 368	232 186	2 477 100	27 988	152 000	43 224 589		278	220	709	88	37	919
1991	2 150 214	818 922	12 604 183	57 500	402 622	211 765 828		521	96	1 511	162	1 282	2 280
1992	2 258	598 819	4 072 328	105 500	12 878 868	1 940 021		21	201	840	1363	2 109	695
1993	262 705	1 330 229	145 851 779	527 873	803 523	474 814		90	940	2 719	174	848	1 379
1994	880 256	190 425	13 686 478	848 229	1 221 641	112 956 142		232	360	2 314	209	1 208	2 448
1995	1 220 225	588 415	58 331 432	3 679 918	1 918 261	127 702 996		428	272	2 888	78	1 935	2 347
1996	493 858	717 921	22 355 210	433 190	1 737 825	156 939 009		136	146	2 428	37	767	4 533
1997	2 019 704	753 881	31 330 468	547 971	2 205 734	7 794 944		639	494	2 528	170	2 641	1 213
1998	2 274 039	486 900	45 196 907	1 534 686	1 260 140	242 910 013		169	554	3 548	285	1 360	4 737
1999	1 210 708	2 438 670	28 900 385	368 424	327 508	115 840 373		357	30 868	1 024	133	153	2 272
2000	5 338 668	442 010	57 315 993	210 006	374 850	10 223 409		1 185	495	2 849	91	16	1 389
2001	2 748 481	760 568	22 054 975	753 858	1 709 513	6 519 111		1 574	265	1 357	122	737	926
2002	897 672	985 422	47 999 183	1 081 417	330 642	116 470 387		382	372	1 152	383	349	1 535
2003	1 074 374	837 462	9 415 977	68 641	1 663 772	156 367 046		356	578	1 604	32	497	843
2004	705 234	641 062	71 797 420	868 169	118 483	42 860 447		216	3603	2 342	54	117	650
2005	566 109	1 031 306	30 464 569	153 518	7 719 199	35 092 108		380	545	2 664	181	1 130	842
2006	1 463 015	814 647	11 136 995	183 379	761 730	15 949 598		1 325	302	2 447	120	898	751
2007	4 475 712	5 878 898	55 133 479	429 457	617 426	112 344 644		745	656	4 635	85	1 118	1 357
2008	943 523	15 848 647	17 699 918	256 435	391 088	9 771 150		495	623	1 932	66	238	653
2009	2 460 622	2 454 724	7 350 598	92 765	273 329	47 102 895		590	351	1 777	132	400	377
2010	4 279 406	4 651 572	14 208 701	388 680	20 533 154	144 732 921		523	1 249	1 559	301	2 410	2 314
2011	1 443 629	5 254 513	25 193 211	39 352	5 418 366	99 098 652		672	1 679	1 941	46	588	1 237
2012	9 167 877	770 043	10 540 815	230 051	5 271 549	38 019 184		786	209	674	225	763	887
2013	1 673 743	1 622 046	8 538 777	1 437 221	2 184 625	16 543 482		632	446	6 989	62	575	1 115
2014	593 818	1 537 037	10 408 785	1 154 026	3 477 012	24 567 410		457	254	1 200	167	854	604
2015	1 846 672	1 427 334	19 559 222	241 022	2 588 226	1 759 266		961	355	1 094	112	527	430
2016	1 089 525	3 704 426	8 748 970	74 742	241 431	65 360 232		646	231	1 819	60	676	965
2017	1 394 494	2 567 331	37 179 509	52 577	123 363	14 302 014		287	350	1 876	25	358	441
2018	2 618 198	586 544	24 524 598	81 294	895 175	5 551 048		678	85	1 057	65	290	706
2019	3 648 255	1 064 920	12 381 937	96 073	11 578 581	6 034 170		837	329	2 746	113	651	473
2020	5 890 694	284 785	9 080 369	189 065	4 253 999	14 654 443		1 045	272	3 070	59	1 160	579
2021	2 096 171	3 760 254	3 956 792	433 166	1 128 779	18 520 741		262	329	1 948	333	777	517
2022	7 024 127	4 064 092	11 186 292	23 052	33 790 206	1 206 293		2 022	901	2 472	10	2 350	307
**Total**	**74 494 354**	**69 146 011**	**890 683 355**	**16 669 245**	**128 352 620**	**2 024 599 380**		**19 927**	**48 630**	**71 713**	**5 543**	**29 819**	**42 721**

WHO: World Health Organization.

Of the 218 353 deaths due to floods, the South-East Asia Region had the highest number (71 713; 32.84%) followed by the Region of the Americas (48 630; 22.27%). The Western Pacific Region had 42 721 deaths (19.57% of all deaths). In the South-East Asia Region, the greatest number of deaths (6989 deaths) occurred in 2013, followed by 4635 deaths in 2007. In the Region of the Americas, the greatest number of deaths (30 868 deaths) occurred in 1999, followed by 3603 deaths in 2004. In the Western Pacific Region, the greatest number deaths (4737 deaths) occurred in 1998, followed by 4533 deaths in 1996 (Table 1).

**Fig. 3:**
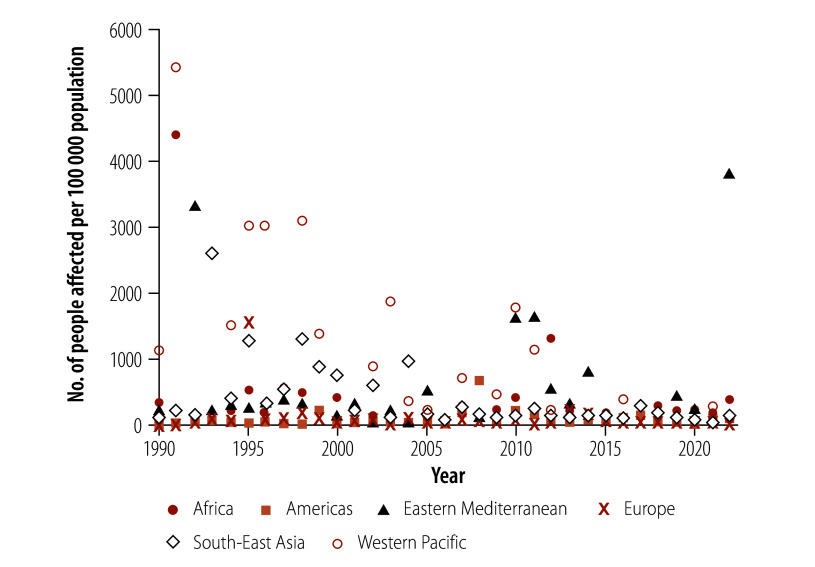
Population affected by natural floods, by year and WHO region, 1990–2022

In 1990, the Western Pacific Region had the highest affected rate of floods (1120.50 per 100 000 population), followed by 335.13 per 100 000 in the African Region and 236.64 per 100 000 in the Eastern Mediterranean Region. The affected rates in the South-East Asia, American and European Regions in 1990 were all lower than 100.00 per 100 000 population. In 2022, the Eastern Mediterranean Region had the highest affected rate (3817.94 per 100 000 population), followed by 383.21 per 100 000 in the African Region. Between 1990 and 2022, the affected rates decreased significantly in the South-East Asia (estimated annual percentage change –5.75%; 95% CI: –9.00% to –2.38%) and Western Pacific (estimated annual percentage change –6.76%; 95% CI: –12.33% to –0.83%) Regions (Table 2). In certain years the affected rates of floods were particularly high in some regions, such as in 1991 in the Western Pacific and African Regions, with 5420.13 per 100 000 and 4407.24 per 100 000 population affected, respectively (Fig. 3).

**Table 2:** Affected population and mortality caused by natural floods in 1990 and 2022 and changes over time, by WHO region

WHO region	Affected			Mortality		
	No. of people per 100 000 population		Estimated annual percentage change (95% CI)	Deaths per 100 000 population		Estimated annual percentage change (95% CI)
	1990	2022		1990	2022	
Africa	335.13	383.21	−0.70 (−4.57 to 3.32)	1.72	1.10	−2.88 (−5.41 to −0.28)
Americas	13.76	88.94	2.33 (−1.09 to 5.88)	0.13	0.20	−2.91 (−7.20 to 1.58)
South-East Asia	60.20	127.46	−5.75 (−9.00 to −2.38)	0.17	0.28	−3.18 (−5.41 to −0.91)
Europe	2.19	5.09	−3.31 (−7.87 to 1.47)	0.07	0.02	−3.53 (−6.50 to −0.47)
Eastern Mediterranean	236.64	3817.94	−0.76 (−6.08 to 4.86)	0.58	2.66	−3.74 (−7.31 to −0.03)
Western Pacific	1120.50	17.41	−6.76 (−12.33 to −0.83)	0.24	0.04	−6.99 (−9.45 to −4.47)

CI: confidence interval; WHO: World Health Organization.

WHO: World Health Organization.

**Fig. 4:**
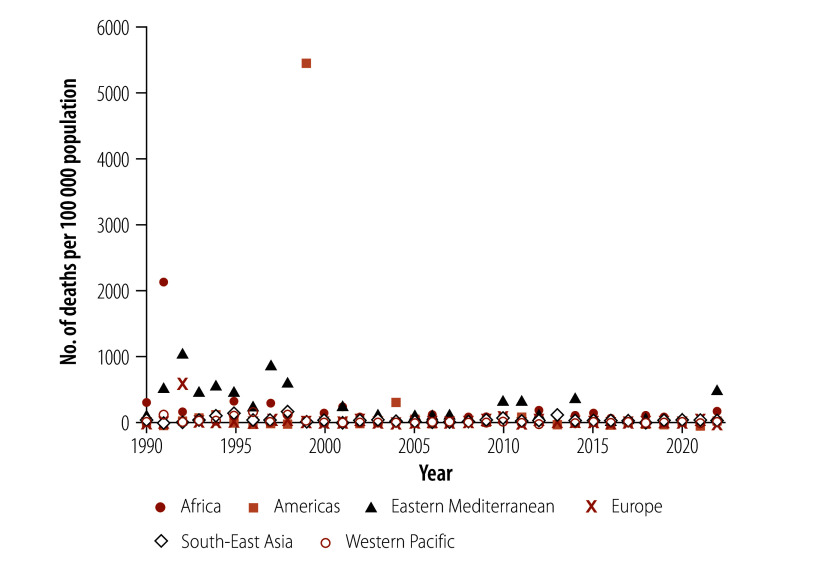
Deaths caused by natural floods, by year and WHO region, 1990–2022

In 1990, the African Region had the highest mortality caused by floods (1.72 per 100 000 population), followed by 0.58 per 100 000 in the Eastern Mediterranean Region (Table 2). In 2022, we estimated the highest mortality in the Eastern Mediterranean Region (2.66 deaths per 100 000 population), followed by 1.10 deaths per 100 000 in the African Region. Between 1990 and 2022, mortality decreased significantly in all WHO regions except the Region of the Americas. The greatest decrease was in the Western Pacific Region (estimated annual percentage change  −6.99%; 95% CI: −9.45% to −4.47%), followed by the Eastern Mediterranean Region (estimated annual percentage change: −3.74%; 95% CI: −7.31% to −0.03%). Mortality was particularly high in 1999 in the Region of the Americas (27.24 per 100 000) and in 1991 in the African Region (10.68 per 100 000; Fig. 4).

WHO: World Health Organization.

### Factors affecting deaths

As shown in Table 3, the proportion of floods with more than 50 deaths decreased from 22.53% (194/861) in 1990–1999 to 12.28% (188/1531) in 2010–2022. From 1990 to 2022, the South-East Asia and Western Pacific Regions had the greatest proportion of floods with more than 50 deaths, 36.20% (236/652) and 23.01% (150/652), respectively. In the South-East Asia Region, 30.37% (236/777) of floods caused more than 50 deaths. Income level was also associated with flood-related deaths. Floods in low- and lower-middle-income countries caused significantly more deaths than in high- and upper-middle-income countries (*P* < 0.001). The proportion of floods with more than 50 deaths was highest for coastal floods (22.67%; 17/75), followed by flash floods (15.40%; 118/766) and riverine floods (14.60%; 363/2487; Table 3).

**Table 3:** Frequency of natural floods by number of deaths caused and associated variables, 1990–2022

Variable	No. (%) of floods				Proportion of > 50 deaths group, %	*P*
	0 deaths or no data (*n* = 1267)	1–9 deaths (*n* = 1403)	10–49 deaths (*n* = 1391)	> 50 deaths (*n* = 652)		
**Time period^a^**						< 0.001
1990–1999	223 (20.67)	197 (16.74)	247 (19.60)	194 (32.55)	22.53	
2000–2009	496 (45.97)	518 (44.01)	492 (39.05)	214 (35.91)	12.44	
2010–2019	360 (33.36)	462 (39.25)	521 (41.35)	188 (31.54)	12.28	
**WHO region**						< 0.001
Africa	276 (21.78)	281 (20.03)	280 (20.13)	93 (14.26)	10.00	
Americas	318 (25.10)	375 (26.73)	273 (19.63)	63 (9.66)	6.12	
South-East Asia	124 (9.79)	161 (11.48)	256 (18.40)	236 (36.20)	30.37	
Europe	310 (24.47)	293 (20.88)	88 (6.33)	17 (2.61)	2.40	
Eastern Mediterranean	69 (5.45)	107 (7.63)	216 (15.53)	93 (14.26)	19.18	
Western Pacific	170 (13.42)	186 (13.26)	278 (19.99)	150 (23.01)	19.13	
**Country income level**						< 0.001
High	278 (21.94)	310 (22.10)	106 (7.62)	11 (1.69)	1.56	
Upper middle	331 (26.12)	342 (24.38)	284 (20.42)	92 (14.11)	8.77	
Lower middle	328 (25.89)	445 (31.72)	564 (40.55)	236 (36.20)	15.00	
Low	330 (26.05)	306 (21.81)	437 (31.42)	313 (48.01)	22.58	
**Flood type**						< 0.001
Coastal	24 (1.89)	17 (1.21)	17 (1.22)	17 (2.61)	22.67	
Flash	146 (11.52)	241 (17.18)	261 (18.76)	118 (18.10)	15.40	
Riverine	649 (51.22)	709 (50.53)	766 (55.07)	363 (55.67)	14.60	
Unknown	448 (35.36)	436 (31.08)	347 (24.95)	154 (23.62)	11.12	

WHO: World Health Organization.

^a^ To ensure that each group had a decade-long span, the three time periods analysed were 1990–1999, 2000–2009 and 2010–2019. Thus, *n* = 1079, 1177, 1260 and 596 for 0 deaths or no data, 1–9 deaths, 10–49 deaths and > 50 deaths, respectively.

The odds of floods with more than 50 deaths decreased significantly over time compared with floods with no deaths or no data. Compared with the African Region, floods in the Eastern Mediterranean Region were more likely to cause 10–49 deaths (aOR: 2.83; 95% CI: 2.04 to 3.94) or > 50 deaths (aOR: 3.85; 95% CI: 2.55 to 5.83). Similar findings were observed in the South-East Asia Region (aOR: 1.87; 95% CI: 1.40 to 2.49 for 10–49 deaths; aOR: 6.72; 95% CI: 4.74 to 9.62 for > 50 deaths) and the Western Pacific Region (aOR: 1.79; 95% CI: 1.33 to 2.41 for 10–49 deaths; aOR: 4.33; 95% CI: 2.94 to 6.44 for > 50 deaths). Compared with high-income countries, the odds of more than 50 deaths from floods were significantly higher for all other incomes levels, particularly for low-income countries, (aOR: 14.34; 95% CI: 7.46 to 30.04). Compared with coastal floods, the odds of causing 10–49 deaths were higher for flash floods (aOR: 3.16; 95% CI: 1.59 to 6.41) and riverine floods (aOR: 2.16; 95% CI: 1.12 to 4.26). No significant differences were seen between flood type in causing > 50 deaths (Table 4).

**Table 4:** Multivariable logistic regression analysis of factors associated with natural flood-related deaths, 1990–2022

Characteristic	aOR (95% CI)		
	1–9 deaths	10–49 deaths	> 50 deaths
**Time period**			
1990–1999	Reference	Reference	Reference
2000–2009	1.13 (0.89 to 1.43)	0.70 (0.55 to 0.90)	0.41 (0.30 to 0.57)
2010–2019	1.39 (1.09 to 1.78)	1.11 (0.86 to 1.43)	0.65 (0.47 to 0.91)
**WHO region **			
Africa	Reference	Reference	Reference
Americas	1.01 (0.75 to 1.35)	1.05 (0.78 to 1.42)	1.25 (0.79 to 1.98)
Eastern Mediterranean	1.32 (0.93 to 1.89)	2.83 (2.04 to 3.94)	3.85 (2.55 to 5.83)
Europe	0.77 (0.57 to 1.04)	0.36 (0.25 to 0.50)	0.34 (0.18 to 0.61)
South-East Asia	1.02 (0.74 to 1.41)	1.87 (1.40 to 2.49)	6.72 (4.74 to 9.62)
Western Pacific	0.86 (0.63 to 1.18)	1.79 (1.33 to 2.41)	4.33 (2.94 to 6.44)
**Income level**			
High	Reference	Reference	Reference
Upper middle	0.85 (0.67 to 1.07)	2.05 (1.53 to 2.76)	7.05 (3.74 to 14.54)
Lower middle	1.10 (0.86 to 1.41)	2.89 (2.15 to 3.90)	8.96 (4.81 to 18.32)
Low	0.74 (0.54 to 1.00)	2.31 (1.65 to 3.25)	14.34 (7.46 to 30.04)
**Flood type**			
Coastal	Reference	Reference	Reference
Flash	2.02 (1.04 to 4.01)	3.16 (1.59 to 6.41)	2.02 (0.94 to 4.41)
Riverine	1.45 (0.77 to 2.80)	2.16 (1.12 to 4.26)	1.50 (0.73 to 3.14)
Unknown	1.18 (0.62 to 2.31)	1.39 (0.71 to 2.78)	0.87 (0.41 to 1.87)

aOR: adjusted odds ratio; CI: confidence interval; WHO: World Health Organization.

Note: The comparison is 0 deaths or no data during a flooding event for all the three death groups. For example, low-income countries have a higher likelihood of experiencing more than 50 deaths instead of 0 deaths than high-income countries.

## Discussion

Floods have affected billions of people over the past 30 years and resulted in hundreds of thousands of deaths in the 168 countries included in our study. China, India, United States of America and Bangladesh, in that order, were the most affected countries. Of the WHO regions, the South-East Asia and Western Pacific Regions had the largest number of people affected and killed by floods. However, the Eastern Mediterranean and African Regions had the highest number of people affected and killed by floods per 100 000 population in 2022. Furthermore, countries with lower income level had a higher risk of deaths caused by floods. Our findings underscore the urgent need to manage risks and enhance preparedness measures for effective responses to floods. 

China was the country most affected by natural floods, with the highest number of people affected and second-highest number of deaths. Despite significant investments in flood defences following the 1998 floods, urban flooding remains a serious concern, with more than 157 cities affected since 2006.^14^^,^^15^ Catastrophic floods in Beijing (2012) and Zhengzhou (2021) underscored the need for adaptive traffic management, improved levees, enlarged reservoirs and advanced early-warning systems.^16^^–^^19^ In the United States, floods also pose a great threat to lives and property, and impose a substantial financial burden on the National Flood Insurance Program, which has accrued more than US$ 20 billion in debt since 2005.^20^^,^^21^ Climate change, implicated in escalating precipitation levels, contributes to growing flood-induced damages.^22^ Similarly, Bangladesh and India have faced increasing flood incidences due to climate change, with hasty developmental projects exacerbating the impacts.^23^ Therefore, governments need to use scientific evidence to devise robust flood mitigation strategies, including successful so-called room for the river approaches, which involve enlarging the capacity of rivers and improving overall floodplain management.^24^^,^^25^ Collaboration across state and international borders with relevant stakeholders is critical.

Regionally, large numbers of people in the Western Pacific and South-East Asia Regions are affected by floods. The South-East Asia, American and Western Pacific Regions, in that order of size, accounted for 74.68% (163 064/218 353) of all deaths due to floods. Of note, our study also found that although the Eastern Mediterranean and African Regions had fewer people affected, the population affected and the mortality due to floods in these regions were the highest in 2022. This situation reflects that in low-income countries, although the disaster-affected population might be smaller, the magnitude of the disaster is greater than in other areas. Thus, areas where populations are substantially affected require comprehensive flood reduction strategies that are well planned and executed to mitigate the effects of floods. Regions with high mortality need to gauge flood risks more accurately and improve emergency management of floods and other disasters to reduce fatalities from floods. To achieve this goal might require greater investment in and redistribution of resources for better flood prevention and emergency response systems.

The risk of death from floods was higher in lower-income countries than in high-income countries. Although economic development can increase disaster-related economic losses, improvements in emergency preparedness, response and coping capacity can reduce vulnerability to disasters, thereby lowering the number of affected individuals and disaster-related deaths.^7^ Therefore, economic assistance and policy support for low-income countries are important. Helping these countries to build disaster risk analysis systems could guide the development of strategies for managing disasters, and the identification of vulnerable countries and regions and the factors contributing to social vulnerability.^18^^,^^27^

Our analysis showed notable time trends in the population affected and mortality due to floods, as well as reduced odds of floods resulting in more than 50 deaths. The decreasing trends suggest an improvement in our global, regional and national responses to such disasters, potentially owing to advances in predictive technology, infrastructure and disaster management strategies. However, it is important to remember that, while these declines are promising, flood events are dependent on a variety of fluctuating factors, such as changing climate patterns. Our findings warrant further in-depth investigations and discussion to fully appreciate their complex implications.

Our study had some limitations. Our findings depended on the data source. In several events, the reported number of deaths, injuries and people affected might be lower than the actual situation, and hence the impact of floods might be underestimated. Our study did not focus on personal factors, and future studies that have access to individual-level data might want to consider exploring these dimensions, given the potential influence of personal factors on the impact of floods. This information could provide a more nuanced understanding of how personal factors might modulate the effects of floods.

Our findings highlight the continued serious impact of floods on countries and populations and hence the urgent need for stronger disaster risk governance, effective flood mitigation strategies, improved emergency systems and international aid, especially for low-income countries.
